# Prodromal Markers in Parkinson’s Disease: Limitations in Longitudinal Studies and Lessons Learned

**DOI:** 10.3389/fnagi.2016.00147

**Published:** 2016-06-22

**Authors:** Sebastian Heinzel, Benjamin Roeben, Yoav Ben-Shlomo, Stefanie Lerche, Guido Alves, Paolo Barone, Stefanie Behnke, Henk W. Berendse, Bastiaan R. Bloem, David Burn, Richard Dodel, Donald G. Grosset, Michele Hu, Meike Kasten, Rejko Krüger, Marcello Moccia, Brit Mollenhauer, Wolfgang Oertel, Ulrike Suenkel, Uwe Walter, Karin Wirdefeldt, Inga Liepelt-Scarfone, Walter Maetzler, Daniela Berg

**Affiliations:** ^1^Department of Neurodegeneration, Hertie Institute for Clinical Brain Research, University of TübingenTübingen, Germany; ^2^German Center for Neurodegenerative Diseases, University of TübingenTübingen, Germany; ^3^School of Social and Community Medicine, University of BristolBristol, UK; ^4^Norwegian Centre for Movement Disorders and Department of Neurology, Stavanger University HospitalStavanger, Norway; ^5^Center for Neurodegenerative Diseases (CEMAND), Neuroscience Section, Department of Medicine, University of SalernoSalerno, Italy; ^6^Department of Neurology, University of HomburgHomburg, Germany; ^7^Department of Neurology and Neuroscience Campus Amsterdam, VU University Medical CentreAmsterdam, Netherlands; ^8^Radboud University Medical Center, Donders Institute for Brain, Cognition and Behavior, Department of NeurologyNijmegen, Netherlands; ^9^Institute of Neuroscience, Newcastle UniversityNewcastle Upon Tyne, UK; ^10^Department of Neurology, Philipps-University MarburgMarburg, Germany; ^11^Institute of Neurological Sciences, Queen Elizabeth University HospitalGlasgow, UK; ^12^Oxford Parkinson’s Disease Centre and Nuffield Department of Clinical Neurosciences, University of OxfordOxford, UK; ^13^Institute of Neurogenetics, University of LübeckLübeck, Germany; ^14^Clinical and Experimental Neuroscience, Luxembourg Center for Systems BiomedicineBelva, Luxembourg; ^15^Paracelsus-Elena-KlinikKassel, Germany; ^16^Department of Neuropathology, University Medical CenterGöttingen, Germany; ^17^Department of Neurology, University of RostockRostock, Germany; ^18^Department of Medical Epidemiology and Biostatistics and Department of Clinical Neuroscience, Karolinska InstitutetStockholm, Sweden; ^19^Department of Neurology, Christian-Albrechts-UniversityKiel, Germany

**Keywords:** Parkinson’s disease, prodromal, cohort, prospective, case-control, clinical, longitudinal, marker

## Abstract

A growing body of evidence supports a prodromal neurodegenerative process preceding the clinical onset of Parkinson’s disease (PD). Studies have identified several different prodromal markers that may have the potential to predict the conversion from healthy to clinical PD but use considerably different approaches. We systematically reviewed 35 longitudinal studies reporting prodromal PD features and evaluated the methodological quality across 10 different predefined domains. We found limitations in the following domains: PD diagnosis (57% of studies), prodromal marker assessments (51%), temporal information on prodromal markers or PD diagnosis (34%), generalizability of results (17%), statistical methods (accounting for at least age as confounder; 17%), study design (14%), and sample size (9%). However, no limitations regarding drop-out (or bias investigation), or report of inclusion/exclusion criteria or prodromal marker associations were revealed. Lessons learned from these limitations and additional aspects of current prodromal marker studies in PD are discussed to provide a basis for the evaluation of findings and the improvement of future research in prodromal PD. The observed heterogeneity of studies, limitations and analyses might be addressed in future longitudinal studies using a, yet to be established, modular minimal set of assessments improving comparability of findings and enabling data sharing and combined analyses across studies.

## Introduction

**Figure 1:**
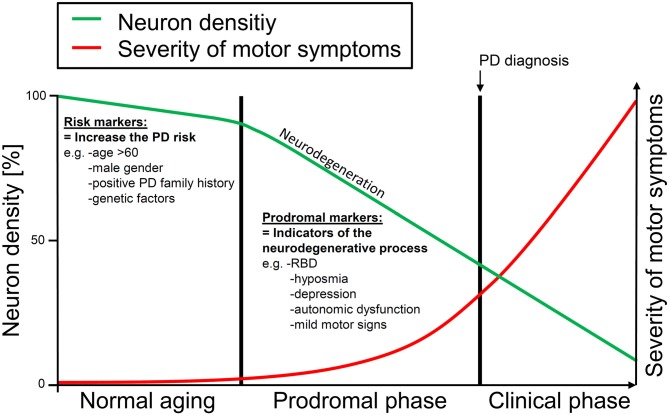
**Graphical illustration of the prodromal phase with early neurodegenerative changes occurring years or even decades before the clinical diagnosis of Parkinson’s disease (PD) can be made.** Risk markers may increase the PD risk without directly being associated with neurodegenerative changes. Only slight loss of neurons due to the normal aging process can be detected in this phase. Prodromal markers represent indicators of the early neurodegenerative process in which neuronal loss is accelerated. This phase may ultimately lead to PD. Risk markers increase the likelihood that an individual may enter the prodromal phase and may finally develop motor PD. Prodromal markers may indicate the ongoing neurodegenerative process and may help to identify those individuals who will likely develop PD in the future.

Parkinson’s disease (PD) is the second most prevalent neurodegenerative disorder. PD is characterized by motor features of resting tremor, bradykinesia, rigidity, and postural instability, but also non-motor symptoms (e.g., hyposmia, depression, autonomic dysfunction) are common in PD patients (Chaudhuri et al., 2006; Jankovic, 2008). However, by the time motor symptoms allow a clinical diagnosis of PD an extensive loss of dopaminergic nigrostriatal neurons will already have taken place due to an ongoing neurodegenerative process spanning years or even decades (Bernheimer et al., 1973; Marek et al., 2001). Several clinical markers have been suggested to indicate this early “prodromal” progressive degenerative process preceding the clinical diagnosis of PD (Gonera et al., 1997; Lang, 2011; Stern et al., 2012). Such prodromal markers include motor as well as non-motor symptoms. For instance, subtle motor signs of the cardinal motor symptoms of PD have been shown to be more frequent in incident PD patients than in controls (Berg et al., 2013b). In addition, a number of non-motor symptoms such as constipation, urinary dysfunction (Schrag et al., 2015), olfactory dysfunction (Ross et al., 2008), depression, anxiety (Schrag et al., 2015), and sleep disorders (Gao et al., 2011b; Postuma et al., 2015), have been shown to occur before the diagnosis of PD (see Figure 1 for a graphical illustration of the concept of prodromal PD). Importantly, prodromal markers of PD could enable an early detection of PD and possibly more effective or even preventive early treatments of the disease.

While numerous longitudinal cohort studies support the concept of prodromal PD and prodromal markers (e.g., Abbott et al., 2001; Postuma et al., 2015; Schrag et al., 2015), the comparability of study results is hampered by methodological differences (Lerche et al., 2015), such as design, type of assessments and analytical methods. Furthermore, limitations of individual studies might additionally affect comparability and interpretation of findings. However, limitations in these prodromal marker studies have not yet been systematically reviewed. Therefore, we performed a systematic review of published longitudinal studies investigating prodromal markers in PD to examine the methodological quality of these studies and to identify key areas that could be improved in future research.

## Materials and Methods

### Search Criteria

This systematic review focuses on longitudinal studies in the prodromal phase of PD. We defined *prodromal markers* as indicators of an ongoing neurodegenerative process in the central or peripheral nervous system prior to the typical symptoms allowing a clinical diagnosis, in our case PD. In contrast, *risk factors* are associated with an increased risk of developing PD, which themselves are not a sign of an ongoing neurodegenerative process and may never lead to it (e.g., pesticide exposure).

We acknowledge that the attribution of a factor to risk and/or prodromal marker categories is sometimes difficult and disputed (e.g., smoking habit). We therefore decided to use a pragmatic approach based on the definitions used by the study authors themselves, rather than impose our own definition.

We searched the literature following a two step-approach in order to identify studies reporting prodromal marker associations preceding the diagnosis of PD. First, we employed a PubMed database search (November 2014) for articles in English using the search term: “prodromal” and “PD” and “longitudinal”. Second, we performed a MEDLINE search (October 2015) for publications missed so far, which identified studies using the terms “prediagnostic”, “preclinical”, “premotor” or “prodromal”, and “Parkinson” (for query details see the “Supplementary Material”).

We disregarded markers of the “nascent sciences” (e.g., protein levels), neuro-imaging except transcranial sonography (TCS), which can be very easily applied in neurodegenerative disorders, or behavioral habits that could be due to personality traits (e.g., smoking). Inclusion criteria comprised: (1) studies providing data with the endpoint of new onset PD, i.e., the conversion from healthy to PD, were considered; (2) assessment of at least one prodromal symptom at the baseline investigation; and (3) for multiple publications of the same prodromal marker in the same cohort, the study with the largest sample size or, if equal, the longest follow-up was selected. We excluded: (a) study designs other than longitudinal (e.g., cross-sectional); (b) studies focusing on risk factors; (c) studies in which the majority of converters developed parkinsonism other than idiopathic PD; (d) PD treatment/intervention studies; (e) non-human studies; and (f) review articles, book chapters, editorials, commentaries, hypothesis articles, meta-analyses, and abstracts.

The initial PubMed search listed 292 publications. Of these, 251 were excluded after screening of abstracts for the exclusion criteria above. Here, often multiple reasons for exclusion applied (e.g., molecular marker, cross-sectional, non-human) and we therefore resigned from describing the individual reasons for exclusion of the respective studies in detail. After full text screening of 41 articles, 14 additional publications were excluded while 28 articles were found to be eligible for inclusion in the review. The MEDLINE search listed 1144 publications and after abstract screening seven additional articles were identified that had been missed in the initial PubMed search. Full text screening of these seven articles did not lead to further exclusions. In total, 35 eligible publications of longitudinal studies investigating prodromal markers in PD were included and systematically reviewed for limitations. However, the evaluation of specific prodromal marker associations or their comparison between studies was not in the scope of this systematic review.

### Limitations of Studies

A specific set of criteria or limitations to be considered for the evaluation of longitudinal studies in prodromal PD has not been established. Yet, for the interpretation of reported prodromal marker associations, numerous and partly marker specific aspects may have to be considered. As a starting point, we used evidence-level criteria (Edlund et al., 2004; Maetzler et al., 2009; originally designed by the American Academy of Neurology (AAN) for intervention study evaluation) comprising five more generic aspects. We complemented this list of limitation categories by methodological and informative aspects more specific to longitudinal studies in prodromal PD. In total, 10 different limitation categories were defined:

(1) Study design: we regarded prospective cohort studies, historical cohort and “nested” case-control studies as the most valid study designs, where participants belonged to an explicit cohort and prodromal marker data had been already collected prior to disease onset. Conventional case-control studies or other designs were regarded as methodologically weaker and therefore fulfilled the criterion for this limitation. We also indicated whether studies were population-based or clinical cohorts, but we did not include this aspect in the evaluation of limitations.

(2) No clearly stated inclusion or exclusion criteria; (3) no adequate accounting for drop-outs (or investigation of potential drop-out bias) in studies with less than 80% of enrolled subjects at follow-up; (4) insufficient number of participants (for prospective cohort studies, historical cohort and “nested” case-control studies: *n* < 50 at baseline, for retrospective and conventional case-control studies: *n* < 100); (5) no presentation of effects indicated by odds ratio (OR), relative risk (RR), hazard ratio (HR), likelihood ratio (LR), sensitivity/specificity, or lack of clear descriptive results from which such effects could be calculated. Mere *t*-test results or reported *P*-values were considered insufficient.

We considered five additional limitation criteria, which are specifically relevant for the evaluation of reported prodromal marker associations; (6) diagnosis of idiopathic PD by non-specialists in movement disorders, only probable PD diagnosis or uncertainties regarding the parkinsonian disease status in “healthy” individuals at baseline; (7) limitations in prodromal marker assessments (e.g., self-reports, marker diagnosis by non-specialists); (8) reduced generalizability of findings (e.g., only males); (9) limitations of statistical analyses, e.g., no appropriate accounting for confounders (at least: age) in the design or analysis; and (10) absent or limited data on the temporal sequence, e.g., duration of marker presence at baseline, or latency period between exposure measurement and onset of PD.

## Results

### Descriptive Results

The 35 reviewed studies assessed the following prodromal markers: five studies investigated sleep disturbances (three Rapid eye movement sleep Behavior Disorder (RBD) (Schenck et al., 1996; Iranzo et al., 2014; Postuma et al., 2015), two daytime sleep (Abbott et al., 2005; Gao et al., 2011b)), three hyposmia (Haehner et al., 2007; Ross et al., 2008; Ponsen et al., 2010), three constipation (Abbott et al., 2001; Savica et al., 2009; Gao et al., 2011a), three cardiac function (i.e., cardiac stress test performance (Palma et al., 2013; Yahalom et al., 2014)), cardiac disease (Jain et al., 2012), one cognitive deficits (Sánchez-Ferro et al., 2013), one pain (Lin et al., 2013), one vital exhaustion (Clark et al., 2013), 10 anxiety/depression (three anxiety (Weisskopf et al., 2003; Bower et al., 2010; Lin et al., 2015), five depression (Leentjens et al., 2003; Fang et al., 2010; Shen et al., 2013; Gustafsson et al., 2015; Walter et al., 2015), two anxiety/depression (Ishihara-Paul et al., 2008; Jacob et al., 2010)), one erectile dysfunction (Gao et al., 2007), one transcranial sonography substantia nigra hyperechogenicity (TCS SN+; Berg et al., 2013a), and six studies (Gonera et al., 1997; Gaenslen et al., 2011; Berg et al., 2013b; Lerche et al., 2014; Plouvier et al., 2014; Schrag et al., 2015) assessed several prodromal markers or marker combinations without a primary focus on a particular prodromal marker.

Of the 35 studies, 28 (80%) comprised population-based samples or medical register data and 7 (20%) clinical cohorts. Study details including study design, markers, sample size, temporal information, prodromal marker effects, and limitations are given in the Supplementary material.

### Limitations in Studies on Prodromal Markers in PD

#### Study Design

Five (14%) studies showed limitations in study design (two retrospective studies (Shen et al., 2013; Lin et al., 2015) and three conventional case-control studies (Gonera et al., 1997; Jacob et al., 2010; Gaenslen et al., 2011)). The other studies were prospective cohort studies (*n* = 22; 63%; Schenck et al., 1996; Abbott et al., 2001, 2005; Weisskopf et al., 2003; Gao et al., 2007, 2011a,b; Haehner et al., 2007; Ishihara-Paul et al., 2008; Ross et al., 2008; Bower et al., 2010; Ponsen et al., 2010; Jain et al., 2012; Berg et al., 2013a,b; Clark et al., 2013; Lin et al., 2013; Sánchez-Ferro et al., 2013; Iranzo et al., 2014; Lerche et al., 2014; Postuma et al., 2015; Walter et al., 2015), or nested case-control studies (*n* = 8; 23%; Leentjens et al., 2003; Savica et al., 2009; Fang et al., 2010; Palma et al., 2013; Plouvier et al., 2014; Yahalom et al., 2014; Gustafsson et al., 2015; Schrag et al., 2015).

#### No Inclusion/Exclusion Criteria

No study showed a total lack of inclusion or exclusion criteria. However, criteria differed between studies dependent on the data source (e.g., insurance/medical registers, hospital records, clinical study) and on study-specific inclusion/exclusion criteria as relevant for marker assessments and PD diagnosis.

#### Drop-Outs

No study reported a drop-out rate of over 20% between baseline and last follow-up without investigating factors associated with the drop-out to exclude a potential drop-out bias.

#### Insufficient Sample Size

Three studies (9%) did not fulfill the criterion of sufficient sample size. Two prospective cohorts (Schenck et al., 1996; Haehner et al., 2007) with <50, and one case-control study (Gonera et al., 1997) with <100 participants at baseline.

#### No Report of Marker Associations

No study failed to report statistical effects or detailed descriptive statistics.

#### Reduced Validity of PD Diagnosis

Eighteen studies (51%; Gonera et al., 1997; Leentjens et al., 2003; Weisskopf et al., 2003; Gao et al., 2007, 2011a; Ishihara-Paul et al., 2008; Ross et al., 2008; Savica et al., 2009; Bower et al., 2010; Fang et al., 2010; Jacob et al., 2010; Jain et al., 2012; Clark et al., 2013; Lin et al., 2013, 2015; Sánchez-Ferro et al., 2013; Gustafsson et al., 2015; Schrag et al., 2015) showed diagnostic limitations, as the diagnosis was not explicitly made by a movement disorder specialist. Alternatively, PD diagnosis was based on disease classification codes, self-reports, use of PD medication (e.g., levodopa) and/or insurance/medical registers, or death certificate documentation. Two additional studies (6%; Haehner et al., 2007; Walter et al., 2015) included “healthy” individuals with borderline PD motor symptoms as indicated by Unified PD Rating Scale part III (UPDRS-III) scores. Here, a UPDRS-III borderline score was defined as 5 or more (Haehner et al., 2007), or individuals with UPDRS-III > 9 (but without definite PD diagnosis) were also considered “healthy” (Walter et al., 2015).

#### Limitations in Prodromal Marker Assessments

Eighteen studies (51%; Gonera et al., 1997; Abbott et al., 2001, 2005; Leentjens et al., 2003; Weisskopf et al., 2003; Gao et al., 2007, 2011a,b; Ishihara-Paul et al., 2008; Bower et al., 2010; Fang et al., 2010; Jacob et al., 2010; Gaenslen et al., 2011; Lin et al., 2013, 2015; Lerche et al., 2014; Gustafsson et al., 2015; Schrag et al., 2015) showed limitations in prodromal marker assessments. Marker information was based on questionnaires/self-reports in 13 studies with 2 studies (Gao et al., 2007; Gaenslen et al., 2011) using longer term retrospective self-reports with possible recall bias, and was based on medical records (e.g., diagnosis of depression in primary care) in five studies (Gonera et al., 1997; Leentjens et al., 2003; Fang et al., 2010; Gustafsson et al., 2015; Schrag et al., 2015).

#### Reduced Generalizability of Findings

Findings of six studies (17%; Schenck et al., 1996; Abbott et al., 2001, 2005; Weisskopf et al., 2003; Gao et al., 2007; Ross et al., 2008) might not be generalizable to females as only males were investigated.

#### Limitations of Statistical Analyses

Six studies (17%; Schenck et al., 1996; Haehner et al., 2007; Berg et al., 2013a,b; Lerche et al., 2014; Walter et al., 2015) did not account for potentially confounding age effects in their statistical analysis or design. Many studies additionally accounted for a number of other confounders as relevant for PD, elderly individuals and/or prodromal marker interpretation in the statistical analysis or via appropriate exclusion criteria.

#### Temporal Uncertainties

Twelve studies (34%; Gao et al., 2007, 2011a,b; Haehner et al., 2007; Ishihara-Paul et al., 2008; Jain et al., 2012; Berg et al., 2013a,b; Sánchez-Ferro et al., 2013; Shen et al., 2013; Lerche et al., 2014; Plouvier et al., 2014) did not report any temporal information on the duration of marker presence at baseline, PD conversion (relative to baseline), or PD incidence (in person-years) in presence/absence of prodromal markers, in addition to the mere time-span between baseline and last follow-up.

**Figure 2:**
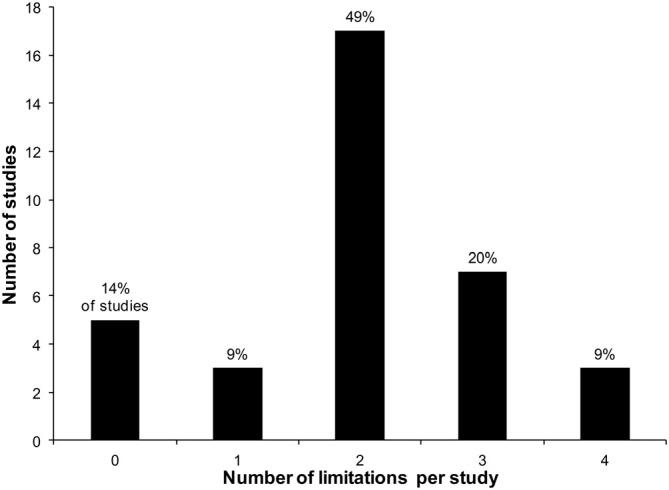
**Frequency of limitations in prodromal PD studies**.

Overall, five studies (14%; Ponsen et al., 2010; Palma et al., 2013; Iranzo et al., 2014; Yahalom et al., 2014; Postuma et al., 2015) showed no limitation according to our criteria. However, most studies (*n* = 30, 86%) showed one or more limitations out of the 10 proposed categories. Limitations in two categories per study [median; mean (SD): 1.7 (1.1) limitations] were most frequently found. Figure 2 shows how many of the reviewed studies had 0, 1, 2, 3 or 4 limitations in the 10 proposed limitation categories.

**Figure 3:**
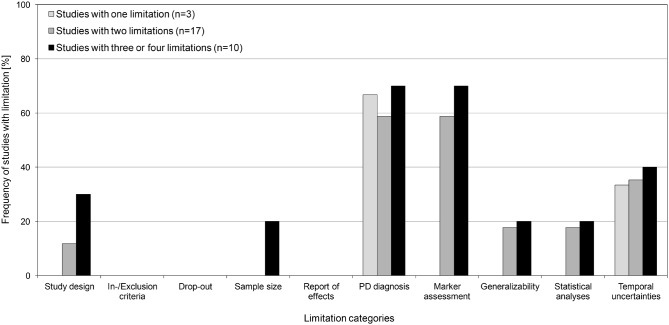
**Frequencies (in %) of studies with a limitation in the 10 predefined limitation categories.** Frequencies are shown for studies with, in total, 1 limitation, 2 limitations and studies with 3 or more limitations.

For studies with 1, 2 and those with 3 or more limitations, the frequency [%] of studies with limitations is shown for each of the predefined limitation categories (Figure 3). Only studies with ≥3 limitations showed limitations in sample size and, compared to studies with fewer limitations, more frequently showed limitations in all other categories (if at all observed for that category). However, marked differences were only observed for limitations in study design, which were more than twice as frequent in studies with ≥3 limitations (30%) compared to studies with 2 limitations (12%). Studies with 1 limitation only showed limitations in PD diagnosis or temporal information.

## Discussion—Lessons Learned

In this systematic review we set out to identify and categorize limitations of longitudinal studies that were designed to examine potential prodromal features of PD. We aimed to set the frame for a critical evaluation of prodromal markers in PD considering limitations as well as individual strengths of studies and to provide recommendations for the improvement of ongoing and future longitudinal studies. We acknowledge the great efforts in each of the reviewed longitudinal studies, which were designed when little was known about prodromal PD.

We defined 10 limitation categories for prodromal longitudinal studies based on evidence-level-criteria (Edlund et al., 2004; Maetzler et al., 2009) as well as additional aspects important for the evaluation of prodromal marker associations. While five studies (14%) showed no limitation in the proposed categories, 30 studies (86%) showed 1 or more limitations suggesting that future studies and reporting of findings can be improved.

Importantly, limitations can introduce different types of bias (e.g., non-differential or differential) which can either attenuate or enhance associations so that the observed associations may be either an under- or over-estimate of the “true” effects. The different limitation categories are discussed below.

### 

#### Study Design (14% of Studies)

Two retrospective cohort and three conventional case-control studies were considered methodologically weaker compared to prospective or nested case-control studies. These designs may be more susceptible to bias in data selection/analysis, and confounders may be more likely to go unrecognized. Thereby, associations rather than true causal relationships between prodromal markers and the development of PD might be established.

#### No Inclusion/Exclusion Criteria (0%)

Specifications of inclusion/exclusion criteria varied between studies. While their appropriateness was not assessed, studies should carefully select specific and appropriate criteria to increase the specificity of prodromal marker effects (without introducing a selection bias).

#### Drop-Outs (0%)

Apart from the reduction of sample size, drop-out of participants over time might not be random but associated with other characteristics, e.g., age, sex, or health-related aspects, such as depression or physical mobility. Thus, demographics and factors related to markers or PD diagnosis should be investigated, especially when the drop-out rate is high, e.g., >20%. However, also a small number of (highly biased) drop-outs may alter findings, and potential biases in drop-outs should therefore always be explored. However, drop-out rates cannot always be inferred from the data and factors linked to drop-out (or motivational aspects of study participation over years) with potential impact on prodromal marker associations remain elusive and should be further investigated.

#### Insufficient Sample Size (9%)

The criterion of required sample size was relatively liberal and was based on intervention study criteria (Edlund et al., 2004). The lack of specificity of some prodromal markers and/or potential confounders may necessitate a larger sample size to obtain valid and reliable evidence of specific prodromal marker effects. For rare markers (e.g., RBD) with putatively strong associations, smaller sample sizes might suffice than for rather common/unspecific markers with smaller associations. The lack of statistical power due to a small sample size might result in false-negative prodromal marker findings (type II error). However, medical register studies in particular by far exceeded the required sample size.

#### No Report of Marker Associations (0%)

Results should provide statistical effects as ORs, RRs, HRs, LRs or sensitivity/specificity, or at least provide detailed descriptive statistics (of prodromal markers both in PD converters and non-converters). In addition to considering limitations, detailed report of associations is important for comparability of findings and for meta-analyses, which should be maintained as none of the reviewed studies showed limitations in this aspect.

#### Reduced Validity of PD Diagnosis (57%)

PD diagnosis by professionals other than movement disorder specialists or surrogate measures for clinical PD diagnosis (e.g., antiparkinsonian medication) is prone to misdiagnosis (Tolosa et al., 2006). Thereby, the specificity of PD prodromal marker findings might have been reduced due to uncertainties in PD diagnosis in 18 of the reviewed studies (51%). However, many studies of these studies put effort into cross-validation of diagnoses in a sub-sample and/or applied multiple criteria (e.g., repeated PD diagnosis in medical records and PD medication intake) to reduce false-positive PD diagnoses. In two studies (6%) individuals with borderline UPDRS-III scores at baseline were included as healthy subjects, thus, a rather late prodromal phase might have been investigated. Most studies did not report baseline UPDRS-III scores (see below: “Temporal Uncertainties” Section), which should be considered in future publications.

#### Limitations in Prodromal Marker Assessments (51%)

Prodromal markers were frequently assessed using self-reports, interviews, questionnaires or medical record data (with unknown validity of entry or diagnosis, i.e., general practice instead of specialist marker diagnosis). Due to practical aspects quantitative/objective assessments can be difficult to realize for some prodromal markers, in particular, in studies with a large sample size. The degree to which reliability of prodromal markers and evidence of findings is limited may differ between studies and assessment methods. Self-report questionnaires and interviews are prone to retrospective recall bias, deliberate false statements (e.g., erectile dysfunction, depression), or unawareness of marker presence (e.g., hyposmia). Also, subjective marker information may be erroneous due to stress, memory deficits, depression or investigator effects. However, also quantitative/objective information may be erroneous (e.g., diagnosis of depression by general practitioner, olfactory testing when having a cold). Potentially, register data carry the risk of over-diagnosis or less reliable assessment of markers/diseases due to non-specialist diagnosis or insurance/financial reasons affecting data entries. For some markers objective marker information is rarely available (e.g., diagnosis of constipation by gastroenterologist instead of self-reports), and the degree of agreement between quantitative/objective and subjective assessments may vary. For each study and prodromal marker these issues need to be considered. Importantly, in prospective studies a possible bias or marker assessment error likely does not differ between PD converters and non-converters rendering these errors less critical for prodromal marker associations.

#### Reduced Generalizability of Findings (17%)

Studies with reduced generalizability investigated only male cohorts. Some markers are for obvious reasons restricted to a subgroup of individuals (e.g., erectile dysfunction in males). However, other prodromal markers studied in males only would require confirmation in samples containing females.

While not considered for this limitation criterion, for some clinical cohorts and cohorts selectively enriched through recruitment of individuals with prodromal markers, or in at-risk groups (e.g., RBD or genetic risk groups), the relevance and generalizability of findings may need further validation in population-based cohorts. However, even some of the population-based samples are not fully random and specific interactions or biases may need to be considered for these samples.

#### Limitations of Statistical Analyses (17%)

Prodromal marker associations might be modulated by confounders, and for each prodromal marker a different set of confounders might play a role. Advanced age is the most important risk factor for PD and many prodromal markers are more frequent with increasing age. Therefore, statistical analyses or study design should at least account for age as a confounder to increase the specificity of prodromal marker associations. Some studies additionally accounted for a number of other relevant confounders in the statistical analysis (or via appropriate exclusion criteria or matching) that might have increased the specificity of reported prodromal marker associations.

#### Temporal Uncertainties (34%)

For the characterization and evaluation of prodromal marker associations with the conversion from healthy to PD, temporal information is crucial to understand the temporal sequence of progressive neurodegeneration preceding PD diagnosis. This neurodegenerative process and the corresponding association with a prodromal marker may span a (much) longer time than the time span from baseline to PD diagnosis. However, marker duration at baseline is rarely reported and thus it remains elusive to what extent marker duration rather than marker presence influences conversion to PD. All available (temporal) information of prodromal markers and PD diagnosis, as well as motor symptom severity (at each visit) should be reported to advance the understanding of temporal aspects of prodromal motor symptom progression and prodromal markers in PD.

Some limitations are unavoidable, and yet studies with limitations have yielded important information, which might otherwise have been missed. For instance, longitudinal studies with extensive assessment batteries as well as large sample sizes need to use easy-to-apply and quick-to-perform assessments such as questionnaires. Nevertheless, findings from these studies contributed with valuable findings despite the lack of quantitative/objective assessments.

Still, for a correct interpretation of prodromal marker findings and the reported effect sizes, potential limitations should be considered to improve comparability of findings and designs, assessments and analyses of ongoing and future studies in prodromal PD. In this systematic review, the three most frequent limitations were in marker assessments, report/availability of temporal information, and PD diagnosis. These limitations are important to address and in this way may improve future studies of prodromal PD.

In addition to the limitation categories investigated, the review of longitudinal studies in prodromal PD revealed other important aspects to be considered in future research.

Table 1 lists important issues to be addressed in future studies on prodromal markers in PD.

**Table 1:** **Open research questions in prodromal Parkinson’s disease (PD)**.

• What is the temporal sequence of occurrence of prodromal markers and what are characteristic changes in severity/frequency of specific prodromal markers over time?
• How are prodromal markers related to another and to possible confounders (e.g., age, sex)?
• How are differences in prodromal markers between individuals and marker constellations related to the PD risk?
• To what extent are prodromal marker effects generalizable to the general population?
• Is the heterogeneity in (idiopathic) PD symptoms/subtypes already indicated by prodromal markers?
• Can risk/prodromal markers be used for calculations of valid and reliable PD risk probabilities?

### Further Suggestions and Lessons Learned

1. In PD and/or its prodromal phase, markers may not always be present or deteriorate linearly. The key advantage of longitudinal studies is the ability to show the trends in a marker over time, but reversion or fluctuation of marker status over time and its relevance for conversion to clinical PD has not been reported in any of the studies. For continuous markers (e.g., marker severity) and dichotomous markers (present/absent), the longitudinal (possibly non-linear) changes or reversion rate until PD diagnosis may, however, be highly valuable for PD prediction models and future therapeutic strategies in the prodromal phase. From this perspective, it will be of relevance to distinguish between dynamically changing state markers, and stable trait markers (possibly RBD, TCS SN+).
2. Some patients with an initial diagnosis of PD will show additional and/or atypical symptoms that require a re-evaluation of the initial diagnosis. Thus, individuals converting to PD should be clinically investigated over an extended period of time to substantiate the PD diagnosis and to gain further information on subgroups of PD patients and their optimal treatment.
3. Non-significant findings of prodromal markers, sub-group analyses or findings of interactions (with other prodromal markers) have not always been reported, although this information would be valuable especially in a future meta-analysis.
4. Enriched cohorts, i.e., individuals with a relatively increased risk of developing PD due to stratification by prodromal/risk factors (male gender, TCS SN+, positive dopamine transporter scintigraphy, PD relatives), may have a higher PD conversion rate, thus providing greater statistical power for a given sample size or shorter study duration. However, it needs to be considered that for single prodromal marker associations other confounders, e.g., additional risk or prodromal markers, might play a role.
5. It is widely acknowledged that PD is a heterogeneous disease (Foltynie et al., 2002; Erro et al., 2013) and symptom (or sub-symptom) severity, progression rate, as well as many other factors may vary among individual PD patients. This complexity has so far not been addressed by prodromal PD marker studies with a single endpoint of (idiopathic) PD.

### Recommendations for Future Prodromal PD Marker Research

In addition to the present findings and lessons learned from longitudinal studies in the prodromal phase of PD, a large degree of heterogeneity in studies both in the prodromal and clinical phase of PD has been reported (Lerche et al., 2015). This heterogeneity concerns the markers assessed (or not assessed), the assessment tools used, and study design (e.g., age at inclusion, frequency of follow-up), thereby reducing comparability of study findings. Based on this experience, we propose a practical approach to advance prodromal PD marker research.

### Modular Minimal Set of Assessments for Longitudinal Studies in (Prodromal) PD

Ongoing as well as new longitudinal studies may benefit from using a common dataset that is shared with other studies. This formal core dataset should be included in all studies (e.g., demographic data) and can be extended by specific additional modules meeting study resources and needs to answer specific scientific hypotheses. While such a modular minimal set of assessments has not yet been established, two major advantages support this concept. First, when studies are comparable in data quality, replication studies as well as testing of new hypotheses using existing data of published studies promise to be more successful. As each study would still have additional and unique characteristics and sample composition a modular minimal set of assessments would advance our understanding of prodromal PD by improving quality of studies and publications. Second, data sharing for joint analysis would be greatly facilitated by a common data approach. In the future, the proposed cooperative endeavor may provide more valid and reliable prodromal markers (or marker profiles) than single and largely unique studies, and may thereby allow to more accurately investigate the multi-factorial prodromal neurodegenerative process ultimately leading to PD.

## Author Contributions

The authors comprise the members of the BioLoC-PD working group on “Harmonization of biomarker assessment in longitudinal cohort studies in Parkinson’s Disease” of the EU Joint Programme Neurodegenerative Disease Research (JPND). The submitted work has been conceptualized and discussed by all members of this working group. Individual contributions of the authors are as follows: Conception/Discussion of study: SH, BR, YB-S, SL, GA, PB, SB, HWB, BRB, DGG, MH, MK, RK, MM, BM, WO, US, UW, KW, IL-S, WM, DB (JPND BioLoC-PD working group). Search and systematic review methods: SH, YB-S, BR, SL, DB. Literature search: SH, BR, SL, US. Analyses: SH, YB-S, BR, SL. Figures: SH, BR. Drafting of manuscript: SH, BR, YB-S, DB. Revision of manuscript: SH, BR, YB-S, SL, GA, PB, SB, HWB, BRB, DGG, MH, MK, RK, MM, BM, WO, US, UW, KW, IL-S, WM, DB. Supervision: DB.

## Funding

This work of the BioLoC-PD working group was supported by the Federal Ministry of Education and Research (BMBF; funding number: 01ED1410), Germany, under the aegis of the EU Joint Programme—Neurodegenerative Disease Research (JPND). The authors also acknowledge the support by the Deutsche Forschungsgemeinschaft (DFG) and the open access fund of the University of Tübingen.

## Conflict of Interest Statement

The authors declare that the research was conducted in the absence of any commercial or financial relationships that could be construed as a potential conflict of interest. The handling Editor declared a shared affiliation, though no other collaboration, with one of the authors [UW] and states that the process nevertheless met the standards of a fair and objective review.
