# Supplementary material for: Prodromal Markers in Parkinson’s Disease: Limitations in Longitudinal Studies and Lessons Learned
Source: Front Aging Neurosci. 2016 Jun 22;8:147. doi: 10.3389/fnagi.2016.00147 (PMC4916171; doi:10.3389/fnagi.2016.00147)

Supplementary Material

*Prodromal markers in Parkinson's disease: Limitations in longitudinal studies and lessons learned*

**Sebastian Heinzel^*^, Benjamin Roeben, Yoav Ben-Shlomo, Stefanie Lerche, Guido Alves, Paolo Barone, Stefanie Behnke, Henk W. Berendse, Bastiaan R. Bloem, David Burn, Richard Dodel, Donald G. Grosset, Michele Hu, Meike Kasten, Rejko Krüger, Marcello Moccia, Brit Mollenhauer, Wolfgang Oertel, Ulrike Suenkel, Uwe Walter, Karin Wirdefeldt, Inga Liepelt-Scarfone, Walter Maetzler, Daniela Berg**

*** Correspondence: Sebastian Heinzel, PhD: Sebastian.Heinzel@med.uni-tuebingen.de**

**Supplementary Table:** Longitudinal Studies of Prodromal Markers in Parkinson’s Disease Eligible for Inclusion in the Systematic Review.

**Abbreviations:** BL, Baseline; CI, Confidence interval; DLB, Dementia with Lewy bodies; DSM, Diagnostic and Statistical Manual of Mental Disorders; FU, Follow-up; HR, Hazard ratio; HC, Healthy control; MMSE, Mini-Mental State Examination; MSA, Multiple system atrophy; OR, Odds ratio; PD, Parkinson's disease; RBD, Rapid-eye-movement behavior disorder; RR, relative risk; ICD, The International Classification of Diseases; SD, standard deviation; SN+, Substantia Nigra hyperechogenicity; TCS, Transcranial sonography; UKBB, United Kingdom Parkinson's Disease Society Brain Bank; UPDRS, Unified Parkinson's Disease Rating Scale; UPSIT, University of Pennsylvania Smell Identification Test.

| **Study design and sample recruitment** | **Marker** | **Assessment** | **Sample size, number of PD converters, time to conversion** | **Temporal information** e.g. Marker duration at baseline, time to conversion after baseline, incidence per person-years | **Associations of prodromal markers for the conversion of healthy to PD** | **Limitations** | **Study acronym** | **Reference** |
| --- | --- | --- | --- | --- | --- | --- | --- | --- |
| Prospective; Clinical cohort | RBD | Video-polysomnography | BL = 174 RBD FU = 168 RBD 22 PD at FU | 14 years of FU; Marker duration at BL: about 12 years conversion to PD after 7.5 years (median) |  |  |  | (Iranzo et al., 2014) |
| Prospective; Clinical cohort | RBD (Additional assessments of: hyposmia, deficits in color vision, quantitative motor tests, antidepressant use, Lifetime depression/ anxiety, personality or autonomic dysfunction) | Polysomnography, UPSIT, Farnsworth-Munsell 100-Hue color test, Timed up-and-go, Purdue pegboard, alternate tap test, UPDRS-III, Tridimensional Personality Questionnaire (TPQ), Autonomic: orthostatic, urinary, erectile, bowel symptoms from the MSA rating scale | BL = 95 RBD FU = 89 RBD (of which 41 developed synucleinopathy: 20 with parkinsonism, 17 PD, 3 MSA; and 21 with dementia (of which 18 had ≥1 cardinal parkinsonism manifestation, 11 met full UKBB criteria for parkinsonism, 3 with abnormal quantitative motor testing, suggesting DLB. | 10 years of FU; Marker duration at BL: 9.2 years (mean), Conversion to parkinsonism/ dementia after 3.8 years (mean) | Color vision: HR = 3.1 (1.5–6.3), Olfaction: (MSA excluded): HR = 2.8 (1.3–6.0), Motor testing (2/4 measures): HR = 3.9 (1.9–8.0), Antidepressant use: HR = 0.29 (0.12–0.68) Lifetime depression/anxiety: non-significant TPQ domains: non-significant, Abnormal autonomic (2/4 measures), %: non-significant | (Associations may not be fully PD-specific as patients in part had other synucleinopathies) |  | (Postuma et al., 2015) |
| Prospective; Clinical cohort | RBD | Polysomnography | BL = 29 RBD FU = 26 RBD of which 13 developed PD | 16 years of FU; From RBD onset to RBD onset to parkinsonism/dementia onset: 14.2 years (mean) |  | Small sample size; only males; statistical analyses not accounting for (potential) age differences between PD converters and non-converters. |  | (Schenck et al. 1996) |
| **Study design and sample recruitment** | **Marker** | **Assessment** | **Sample size, number of PD converters, time to conversion** | **Temporal information** e.g. Marker duration at baseline, time to conversion after baseline, incidence per person-years | **Associations of prodromal markers for the conversion of healthy to PD** | **Limitations** | **Study acronym** | **Reference** |
| Prospective; Population-based | Excessive daytime sleepiness | Excessive daytime sleepiness questionnaire | BL = 3,078 HC (after excluding 61 with PD, 215 with dementia and 378 with missing EDS data) FU = 3,078 of which 43 developed PD | Age-adjusted incidence per 10,000 person-years: with EDS: 55.3 (PD diagnosis 7 months and 4.9 years into FU) without EDS: 17.0  (PD diagnosis 2 months to 7.3 years into FU) | OR = 2.8 (1.1-6.4) OR = 3.3 (1.1-6.4) adjusted for sleep-related features | Only males; marker uncertainty (questionnaire/self-report) | HAAS | (Abbott et al., 2005) |
| Prospective; Population-based | Daytime napping | Self-report | BL / FU = 214,655 (of which 770 PD at FU) | 3-4 years of FU (in prediagnostic PD group) Typical sleeping habit over last 12 months. | For ≥1h daytime napping (and ≥7h nighttime sleep): OR = 1.5 (1.2-1.9) | Marker uncertainty ( self-report); temporal uncertainty | NIH-AARP Diet and Health Study | (Gao et al., 2011b) |
| Prospective; Clinical cohort | Hyposmia | Sniffin’ Sticks | BL = 30 hyposmics  FU = 24 hyposmics (of which 1 PD at FU) | 4 years of FU; unknown marker duration of time until conversion |  | Diagnosis uncertainty (Inclusion of borderline UPDRS-III at BL); small sample size; temporal uncertainty; statistical analyses not accounting for (potential) age differences between PD converters and non-converters. |  | (Haehner et al., 2007) |

| **Study design and sample recruitment** | **Marker** | **Assessment** | **Sample size, number of PD converters, time to conversion** | **Temporal information** e.g. Marker duration at baseline, time to conversion after baseline, incidence per person-years | | **Associations of prodromal markers for the conversion of healthy to PD** | | **Limitations** | | **Study acronym** | **Reference** |
| --- | --- | --- | --- | --- | --- | --- | --- | --- | --- | --- | --- |
| Prospective; Clinical cohort | Hyposmia | Sniffin’ Sticks | Total BL = 361 Total FU = 354 With clinical examination at FU: BL = 78 with 40 hyposmics, 38 normosmics FU = 74; 5 of 40 hyposmics developed clinical PD, 0 of normosmics | 5 years of FU; Motor symptom onset: 15 months (median) after BL | |  | |  | |  | (Ponsen et al., 2010) |
| Prospective; Population-based | Hyposmia | Brief Smell Identification Test (B-SIT) | BL = 2,267 FU1 = 2,267 FU2 = 1,846 with 35 incident PD at FU1/2 | 24.6/10,000 person-years;  Up to 8 years of FU; Time to diagnosis after BL: 4.0 years (mean) | | OR = 5.2 (1.5-25.6); 1. Quartile 4-y-FU | | Only males; questionnaire based PD diagnosis | | HAAS | (Ross et al., 2008) |
| Prospective; Population-based | Constipation | Bowel frequency interview | BL = 127,668 BL: ≤1 bowel movements/3d n = 8,016 FU = 558 incident PD cases | HPFS: 6 years of FU; NHS: 24 years of FU | | Pooled OR = 3.93 (2.26-6.84) ≤1 bowel movements/3d | | PD diagnosed based on medical records;  marker uncertainty (self-report, interview); temporal uncertainty | | HPFS, NHS | (Gao et al., 2011a) |
| Prospective; Population-based | Constipation | Bowel frequency interview | BL = 6,860 FU = 6,790 with 96 incident PD at FU | 24 years of FU; Time to diagnosis: 12 years (mean); Age-adjusted incidence 18.9/10,000 person-years (1 bowel movement/day); 3.8/10,000 person-years (2/day) | | RR = 2.7 (1.3-5.5) < 1 bowel movements/d | | Only males;  marker uncertainty (self-report, interview) | | HAAS | (Abbott et al., 2001) |
| **Study design and sample recruitment** | **Marker** | **Assessment** | **Sample size, number of PD converters, time to conversion** | **Temporal information** e.g. Marker duration at baseline, time to conversion after baseline, incidence per person-years | | **Associations of prodromal markers for the conversion of healthy to PD** | | **Limitations** | | **Study acronym** | **Reference** |
| Nested case-control; Population-based | Constipation | Medical records/medication | BL = 392 FU = 196 incident PD, 196 matched HC | Marker 0–19 years before diagnosis >20 years before diagnosis Enrollment in medical records-linkage system: 38 years (median) | | OR = 1.77 (1.04-2.98) (not caused by medication) Marker 0–19 years before diagnosis: OR = 1.96 (1.05-3.65) >20 years before index year: OR = 2.49 (1.24-5.01) | | PD diagnosis uncertainty | |  | (Savica et al., 2009) |
| Prospective; Population-based | Cardiovascular function | Electrocardiogram, carotid ultrasound | BL = 5,828 FU = 5,828 with 154 incident PD at FU | 10.4 years (mean) of FU | | Electrocardiographic abnormalities: OR = 1.45 (1.02–2.07) carotid stenosis: OR = 2.40 (1.40–4.09) | | PD diagnosis and date of PD onset uncertainty; temporal uncertainty | | CHS | (Jain et al., 2012) |
| Nested case-control; Clinical cohort | Cardiovascular function | Cardiac stress testing (CST), electrocardiogram | BL = 54 HC; FU = 36 HC, 18 incident PD Matched HC | 10 years of FU PD diagnosis 4.27 years (median) after BL | |  | |  | |  | (Palma et al., 2013) |
| Nested case-control; Population-based cohort | Cardiovascular function | Cardiac stress testing (CST), electrocardiogram | BL = 60 HC; FU = 40 HC, 20 PD Matched HC | 4.64 years (mean) between BL and first motor symptom onset; 1 year between BL and FU (PD diagnosis) | | No significant associations | |  | |  | (Yahalom et al., 2014) |
| Prospective; Population-based | Cognition, rate of cognitive decline | 37-MMSE | BL = 2,450 FU= 2,450 with incident 21 PD at FU | 3.3 years (median) between BL and FU | | No significant associations (after accounting for age and other confounders) | | PD diagnosis uncertainty; temporal uncertainty | | NEDICES | (Sanchez-Ferro et al., 2013) |
| **Study design and sample recruitment** | **Marker** | **Assessment** | **Sample size, number of PD converters, time to conversion** | **Temporal information** e.g. Marker duration at baseline, time to conversion after baseline, incidence per person-years | | **Associations of prodromal markers for the conversion of healthy to PD** | | **Limitations** | | **Study acronym** | **Reference** |
| Prospective; Population-based | Pain | National Health and Nutrition examination Survey (NHANES) | BL = 33,388 FU = 33,388 with 32 incident PD at FU | 3 years (median) between BL and FU; incidence rate of PD per 100,000 person-years: 16 (without pain); 40 (mild pain); 109 (mod./severe pain) | | Moderate/severe pain: HR = 2.88 (1.05–7.86). Non-significant when using more stringent PD case definitions (n = 19 PD cases) | | PD diagnosis uncertainty; marker uncertainty (questionnaire, self-report) | | NHIS | (Lin et al., 2013) |
| Prospective; Population-based | Vital exhaustion, impaired sleep | Vital Exhaustion Scale | BL = 9,955 FU = 9,955 108 incident PD at FU | 14 years (mean) of FU; with No-, 5-year, and 10-year time-lag from pre-motor symptoms | | High vs. low vital exhaustion: No-time-lag: HR = 2.50 (1.28-4.89), impaired sleep: HR = 1.49 (0.87-2.56); 5-year time-lag: HR = 1.54 (0.63–3.75) 10-year time-lag: non-significant | | PD diagnosis/ date of PD onset unknown at hospitalization | | CCHS | (Clark et al., 2013) |
| Prospective; Population-based | Anxiety | Crown-Crisp phobic anxiety index | BL = 35,815 FU = 35,815 with 189 incident PD at FU | 12 years of FU; Person-years of PD incidence for anxiety index 0/1, 2, 3, 4+: 156,520; 62,336; 43,508; 67,136 | | RR = 1.5 (1.0-2.1) | | Only males; PD diagnosis uncertainty; marker uncertainty (questionnaire, self-report) | | HPFS | (Weisskopf et al., 2003) |
| Retrospective; Population-based | Anxiety disorder | ICD (9th revision; Clinical Modification) codes 300, 309.24 and 293.84) or use of anxiolytics ( Anatomical Therapeutic Chemical (*ATC*) Classification System code: N05B) | BL = 174,776 FU = 174,776 with 2258 incident PD at FU | 5.5 years (mean) of FU; Crude incidence rate of PD per 1 million person-days: No: 5.18 Mild: 6.60 Moderate: 7.08  Severe anxiety: 12.33 | | Anxiety: Adjusted HR = 1.38 (1.26-1.51) | | Retrospective study design; PD diagnosis uncertainty; marker uncertainty (questionnaire, self-report) | | Taiwan NHRID system | (Lin et al. 2015) |
| **Study design and sample recruitment** | **Marker** | **Assessment** | **Sample size, number of PD converters, time to conversion** | | **Temporal information** e.g. Marker duration at baseline, time to conversion after baseline, incidence per person-years | | **Associations of prodromal markers for the conversion of healthy to PD** | | **Limitations** | **Study acronym** | **Reference** |
| Prospective; Population-based | Anxiety,  pessimistic/depressive personality trait | Minnesota multiphasic personality inventory | BL = 6,822 FU = 5,816 with 156 incident PD at FU | | 29.2 years (median) of FU; Anxiety 1-3 quartile: 138100 person-years; Anxiety 4th quartile: 43097 person-years | | Anxious: HR = 1.63 (95% CI = 1.16-2.27) Pessimistic in men: HR = 1.92 (95% CI = 1.20-3.07) Neuroticism: HR = 1.54 (95% CI = 1.10-2.16) | | PD diagnosis uncertainty (parkinsonism; medical records); marker uncertainty (questionnaire, self-report) |  | (Bower et al., 2010) |
| Prospective; Clinical cohort | Depression (Major depressive disorder; MDD) (Additional assessment of TCS SN status, cognition, olfaction etc.) | DSM-IV criteria | BL = 57 FU = 46 with 3 incident PD at FU | | 10 years (median) of FU; Mean ± SD duration of MDD at FU: No PD: 3.9 ± 5.4  PD: 16.0 ± 5.3 years | |  | | Diagnosis uncertainty (18% of individuals at BL with UPDRS-III scores >9; but without definite PD diagnosis based on UKBB criteria); statistical analyses not accounting for age differences between PD converters and non-converters. |  | (Walter et al., 2015) |
| Nested case-control; Population-based | Depression | ICD-9/10 codes | BL = 562,406 FU = 562,406 with 421,718 matched HC and 140,688 incident PD at FU | | 6.8 years (median) of FU; Depression HR for PD continuously indicated for 3 months to 25 years after BL. | | Within the first year of depression: OR: 3.2 (2.5–4.1); After 15 to 25 years: OR = 1.5 (1.1–2.0) | | PD diagnosis uncertainty; marker uncertainty (ICD codes) | NPR Sweden | (Gustafsson et al., 2015) |

| **Study design and sample recruitment** | **Marker** | **Assessment** | **Sample size, number of PD converters, time to conversion** | **Temporal information** e.g. Marker duration at baseline, time to conversion after baseline, incidence per person-years | **Associations of prodromal markers for the conversion of healthy to PD** | **Limitations** | **Study acronym** | **Reference** |
| --- | --- | --- | --- | --- | --- | --- | --- | --- |
| Nested case-control; Population-based | Depression | Self-reported diagnosis | BL = 280,950  FU = 280,950 with 992 PD incident PD at FU | Depression diagnosed in 5 year intervals (1985-2000), with PD diagnosed after the year 2000. | Depression diagnosed after 2000 OR = 2.0 (1.6-2.4); in 1995–1999: OR = 2.7, 2.0-3.6); in 1985–1994: OR = 1.6 (1.1-2.3); <1985: OR = 1.7 (1.3-2.3). | PD diagnosis uncertainty (self-reported, medical records); marker uncertainty (self-report) | NIH-AARP Diet and Health Study | (Fang et al., 2010) |
| Retrospective; Population-based | Depression | Medical records/insurance database | BL = 23,180 (4,636 with depression) FU = 23,280 with 163 PD incident PD at FU (match. HC) | 10-year FU | HR = 3.24 (2.36-4.44) | Retrospective study design; temporal uncertainty | LHID 2005 | (Shen et al., 2013) |
| Nested case-control; Population-based | Depression | General practice registry | BL = 32,415 FU = 32,415 with 338 incident PD at FU | Medical records from: 1985 to 2000 First depressive episode to PD diagnosis: 10.1 years (mean) | OR = 2.4 (2.1-2.7) | PD diagnosis uncertainty; marker uncertainty (general practice medical records) |  | (Leentjens et al., 2003) |
| Case-control; Population-based | Depression, anxiety | Self-reported medical information | BL = 773 FU = 773 with 371 incident PD at FU (sibling controls not included) | Depression/anxiety/medication 2, 5, 10, and 20 years before PD diagnosis | Lifetime depression/anxiety: OR = 1.42 (1.01-2.00); e.g. 5-y-OR = 2.21 (1.21-4.04) in PD males; non-significant for females | Case-control study design; PD diagnosis uncertainty; marker uncertainty (self-report) | UCLA PEG | (Jacob et al., 2010) |
| Prospective; Population-based | Depression, anxiety | DSM-IV & Health and Life Experiences Questionnaire (HLEQ) | BL = 20,855 FU = 20,855 (with 175 suspected PD; 43 with neurological records) | 7.9 years of FU 160,725 person-years | Depression: HR = 2.01 (0.95-4.22);  Anxiety: HR = 2.52 (0.78-8.20);MHI-5: HR = 1.28 (0.97-1.68) Neuroticism: HR = 1.33 (0.98-1.79) Extroversion: HR = 1.09 (0.80-1.48) | PD diagnosis uncertainty; marker uncertainty (questionnaire, self-report); temporal uncertainty | EPIC-Norfolk | (Ishihara-Paul et al., 2008) |

| **Study design and sample recruitment** | **Marker** | **Assessment** | **Sample size, number of PD converters, time to conversion** | **Temporal information** e.g. Marker duration at baseline, time to conversion after baseline, incidence per person-years | **Associations of prodromal markers for the conversion of healthy to PD** | **Limitations** | **Study acronym** | **Reference** |
| --- | --- | --- | --- | --- | --- | --- | --- | --- |
| Prospective; Population-based | Erectile dysfunction | Retrospective questionnaire | BL = 32,616  FU = 32,616 with 200 incident PD | 16 years of FU | RR = 3.8 (95% CI = 2.4-6.0) | PD diagnosis uncertainty; marker uncertainty (questionnaire, self-report with possible recall bias); only males;  temporal uncertainty | HPFS | (Gao et al., 2007 ) |
| Prospective; Population-based | SN+ | TCS | BL = 1,847 FU1 = 1,535 FU2 = 1,271 with 21 incident PD at FU2  PRIPS: Drop-out of participants was investigated: higher age, more frequent positive PD history in FU2; sex, SN+ status, no differences compared to FU1 | 3 and 5 years of FU, respectively. | RR = 20.6 (5.6-98.8) | Temporal uncertainty; analyses not accounting for age differences between PD converters and non-converters. | PRIPS | (Berg et al., 2013a) |
| Prospective; Population-based | SN+, mild parkinsonian signs, hyposmia, constipation, depression, PD family history | TCS, UPDRS, Sniffin’ Sticks, Interview | BL = 1,847 FU1 = 1,535 FU2 = 1,276 with 21 incident PD at FU2 | 3 and 5 years of FU, respectively. |  | Marker uncertainty (e.g. constipation self-report), temporal uncertainty (marker duration; time to diagnosis); analyses not accounting for age differences between PD converters and non-converters. | PRIPS | (Lerche et al., 2014) |
| Prospective; Population-based | Subtle motor impairment, SN+, hyposmia, constipation, depression, PD family history | TCS, 12 Sniffin’ Sticks, UDPRS-III | BL = 1,847 FU = 1,535 with 11 incident PD at FU | 3 years of FU | Male: RR = 1.8 (0.4-8.5) SN+: RR = 16.8 (3.4-114.9) hyposmia: RR = 6.5 (1.5-31.3) SMI: RR = 4.8 (1.2-20.4) age > 60 y: RR = 3.3 (0.8-16.2) positive family history: RR = 5.4 (1.3-21.4) | Temporal uncertainty; analyses not accounting for age differences between PD converters and non-converters. | PRIPS | (Berg et al., 2013b) |
| **Study design and sample recruitment** | **Marker** | **Assessment** | **Sample size, number of PD converters, time to conversion** | **Temporal information** e.g. Marker duration at baseline, time to conversion after baseline, incidence per person-years | **Associations of prodromal markers for the conversion of healthy to PD** | **Limitations** | **Study acronym** | **Reference** |
| Nested case-control; Population-based | Tremor, balance impairments, constipation, hypotension, erectile dysfunction, urinary dysfunction, dizziness, fatigue, depression, anxiety | Primary care database | BL (e.g. set 5 years before diagnosis) = 30,313 FU: 25,544 matched HC and 4769 incident PD | Marker association calculations for markers 5 and 10 years, respectively before PD diagnosis | 5 years before diagnosis: Tremor: RR = 13.70 (7.82–24.31),  Balance impairments: RR = 2.19 (1.09–4.16), Constipation: RR = 2.24 (2.04–2.46),  Hypotension: RR = 3.23 (1.85–5.52), Erectile dysfunction: RR = 1.30 (1.11–1.51),  Urinary dysfunction: RR = 1.96 (1.34–2.80), Dizziness: RR = 1.99, 1·67–2·37),  Fatigue: RR = 1.56 (1.27–1.91), Depression: RR = 1.76 (1.41–2.17),  Anxiety: RR = 1.41 (1.09–1.79) 10 years before diagnosis: Tremor: RR = 7.59 (1.11–44.83) Constipation RR = 2.01 (1.62–2.49) | PD diagnosis uncertainty; marker uncertainty (primary care medical records) | THIN | (Schrag et al., 2015) |
| Nested case-control; Population-based | Somatic symptoms, autonomic dysfunction, sleep, depression, dementia, hyposmia | Health care registry | BL= 164 FU = 164 with 86 incident PD at FU (matched HC) | Within 2-years prior to PD diagnosis | Somatic symptoms: OR = 2.45 Constipation: OR = 3.32 Sleep disorders: OR = 6.98 | Temporal uncertainty |  | (Plouvier et al., 2014) |
| Case-control; Population based | Self-perceived non-motor and early motor signs | Telephone interview | FU = 186 with 93 PD patients (matched HC) | Recalled onset of first prodromal symptoms: 10.2 years (mean); Mean age of onset for each non-motor and motor sign indicated for controls and PD patients. |  | Case-control study design; marker uncertainty ( self-report with possible recall bias) |  | (Gaenslen et al., 2011) |
| **Study design and sample recruitment** | **Marker** | **Assessment** | **Sample size, number of PD converters, time to conversion** | **Temporal information** e.g. Marker duration at baseline, time to conversion after baseline, incidence per person-years | **Associations of prodromal markers for the conversion of healthy to PD** | **Limitations** | **Study acronym** | **Reference** |
| Case-control; Population based | Various medical symptoms | Hospital/GP records | BL/FU = 60 PD 58 HC | 10-years before PD diagnosis; symptom occurrence 10, 6, 4, 3, 2, 1 years before PD |  | Case-control study design; PD diagnosis uncertainty; marker uncertainty (medical records); small sample size |  | (Gonera et al., 1997) |

**Supplementary information**: PubMed and MEDLINE search queries

PubMed search strategy (November 2014):

“prodromal” AND “Parkinson’s disease” AND “longitudinal”

(Articles written in English)

MEDLINE literature search strategy:

Database: Ovid MEDLINE(R) <1946 to October Week 1 2015>

1 exp Parkinsonian Disorders/ (63479)

2 parkinson*.ti,ab. (77611)

3 1 or 2 (87578)

4 (prediagnostic or prodromal or preclinical or premotor or pre-diagnostic or pre-motor or pre-clinical).ti,ab. (72269)

5 prodromal symptoms/ (551)

6 4 or 5 (72500)

7 3 and 6 (1696)

8 letter/ (923340)

9 editorial/ (375420)

10 news/ (167927)

11 exp historical article/ (345399)

12 Anecdotes as topic/ (4708)

13 comment/ (625819)

14 case report/ (1783962)

15 (letter or comment$).ti. (99622)

16 animals/ not humans/ (4033465)

17 exp Animals, Laboratory/ (766165)

18 exp Animal Experimentation/ (6959)

19 exp Models, Animal/ (454710)

20 exp rodentia/ (2813218)

21 (rat or rats or mouse or mice).ti. (1125197)

22 or/8-21 (8191919)

23 7 not 22 (1220)

24 limit 23 to English language (1144)

The number in brackets indicate the number of hits, i.e. in total 1144 hits for this search query.


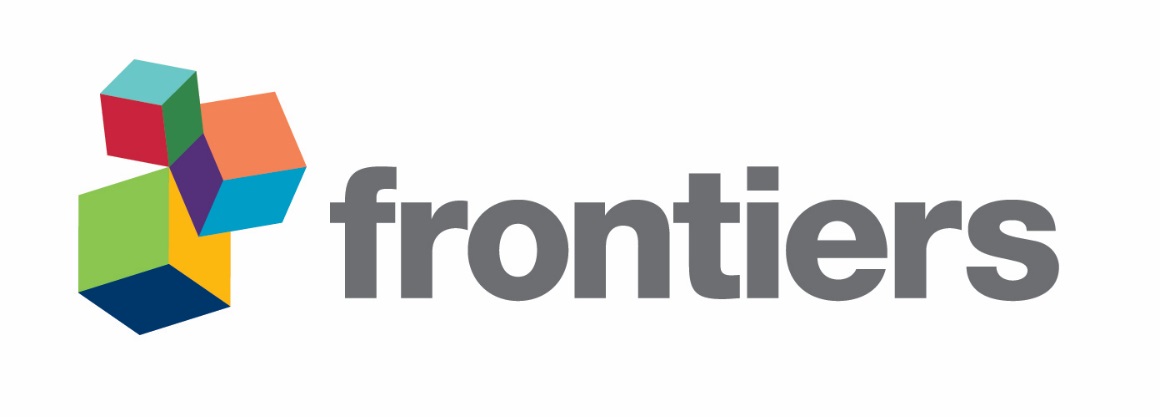

Supplement: Supplementary file 1 [file DataSheet_1.docx]
